# Following Acute Encephalitis, Semliki Forest Virus is Undetectable in the Brain by Infectivity Assays but Functional Virus RNA Capable of Generating Infectious Virus Persists for Life

**DOI:** 10.3390/v10050273

**Published:** 2018-05-18

**Authors:** Rennos Fragkoudis, Catherine M. Dixon-Ballany, Adrian K. Zagrajek, Lukasz Kedzierski, John K. Fazakerley

**Affiliations:** 1 The Roslin Institute and Royal (Dick) School of Veterinary Studies, College of Medicine & Veterinary Medicine, University of Edinburgh, Edinburgh, Midlothian EH25 9RG, UK; rennos.fragkoudis@pirbright.ac.uk (R.F.); cattiwano@yahoo.com (C.M.D.-B.); Adrian.zagrajek@pirbright.ac.uk (A.K.Z.); 2 The School of Veterinary Medicine and Science, The University of Nottingham, Sutton Bonington Campus, Leicestershire LE12 5RD, UK; 3 Department of Microbiology and Immunology, Faculty of Medicine, Dentistry and Health Sciences at The Peter Doherty Institute for Infection and Immunity and the Melbourne Veterinary School, Faculty of Veterinary and Agricultural Sciences, The University of Melbourne, 792 Elizabeth Street, Melbourne 3000, Australia; lukaszk@unimelb.edu.au

**Keywords:** Semliki Forest virus, alphavirus, persistence

## Abstract

Alphaviruses are mosquito-transmitted RNA viruses which generally cause acute disease including mild febrile illness, rash, arthralgia, myalgia and more severely, encephalitis. In the mouse, peripheral infection with Semliki Forest virus (SFV) results in encephalitis. With non-virulent strains, infectious virus is detectable in the brain, by standard infectivity assays, for around ten days. As we have shown previously, in severe combined immunodeficient (SCID) mice, infectious virus is detectable for months in the brain. Here we show that in MHC-II^-/-^ mice, with no functional CD4 T-cells, infectious virus is also detectable in the brain for long periods. In contrast, in the brains of CD8^-/-^ mice, virus RNA persists but infectious virus is not detectable. In SCID mice infected with SFV, repeated intraperitoneal administration of anti-SFV immune serum rapidly reduced the titer of infectious virus in the brain to undetectable, however virus RNA persisted. Repeated intraperitoneal passive transfer of immune serum resulted in maintenance of brain virus RNA, with no detectable infectious virus, for several weeks. When passive antibody transfer was stopped, antibody levels declined and infectious virus was again detectable in the brain. In aged immunocompetent mice, previously infected with SFV, immunosuppression of antibody responses many months after initial infection also resulted in renewed ability to detect infectious virus in the brain. In summary, antiviral antibodies control and determine whether infectious virus is detectable in the brain but immune responses cannot clear this infection from the brain. Functional virus RNA capable of generating infectious virus persists and if antibody levels decline, infectious virus is again detectable.

## 1. Introduction

Most central nervous system (CNS) infections initiated by RNA viruses are clinically acute. Based on infectivity assays in experimental model systems, levels of infectious virus are rapidly reduced to below detectable levels by immune responses. CNS immune responses are highly specialized and regulated [1,2]; the resting CNS, is separated from the blood by the tight endothelial cell junctions of the blood-brain barrier (BBB), it lacks organized lymphoid tissue, has limited antigen presentation capacity [3], limited major histocompatibility complex (MHC) expression [4,5], no antibodies, no functional complement system and an immunosuppressive cytokine environment [6,7]. Virus infection of the CNS can nevertheless give rise to florid immune responses. The CNS is also highly specialised in that it contains many long-lived cells with reduced propensity to undergo apoptosis upon virus infection predisposing, as we have long argued, to virus persistence [8].

Semliki Forest virus (SFV) provides a well-characterised and tractable model of virus encephalitis [9,10]. SFV is efficiently neuroinvasive, allowing study of CNS events without direct intracerebral inoculation and disturbance of the BBB. SFV strains vary in their virulence and can produce acute fatal panencephalitis or non-fatal subacute encephalitis with lesions of inflammatory demyelination. SFV inoculated intraperitoneally first replicates in several tissues resulting in a high titre plasma viraemia from which virus crosses the BBB to establish small perivascular foci of CNS infection and infectious virus is detectable in the brain from day two to ten [11]. SFV predominantly infects neurons and oligodendrocytes, but replication is restricted in the mature neurons of the adult mouse brain [11,12,13]. Immune system clearance of detectable infectious virus is followed by the appearance of lesions of inflammatory demyelination which are dependent upon the presence of CD8^+^ T cells [14]. Following recovery from the acute infection, while infectious virus is no-longer detectable, virus RNA is detectable in the brain for many months [15] and there is continued intrathecal antibody synthesis by plasma cells [16,17]. Similarly, after Sindbis virus (SINV) infection of mice, virus RNA in the brain and intrathecal B-cells and IgG secretion are observed many months after the acute infection has resolved [18,19]. In athymic *nu/nu* mice, which lack T lymphocytes and which produce only anti-viral IgM, titers of infectious SFV in the blood are rapidly reduced to undetectable levels whereas in the brain, high titres of infectious virus remain for months [20]. In SCID mice, with no antibody and no functional T or B cells, high titres of infectious SFV are detectable in both the blood and the brain for several weeks [21]. However, passive transfer of polyclonal anti-SFV antibody rapidly reduces the high levels of infectious virus in the blood and the brain to below the level of detection of the assay. Similarly, a single dose of polyclonal or monoclonal antibody also renders infectious SINV undetectable in the CNS of SCID mice [22].

What remains unclear is whether the alphavirus RNA which persists in the brain can generate infectious virus. Here we show, using passive transfer of antibodies to SFV infected SCID mice and through immunosuppression of aged mice which have recovered from SFV infection, that the viral RNA which persists in the mouse brain is capable or regenerating infectious virus.

## 2. Materials and Methods

### 2.1. Virus

The avirulent A7(74) strain of Semliki Forest virus was used in this study [11]. All mice were inoculated intraperitoneally (i.p.) with 5 × 10^3^ pfu of virus in 0.1 mL PBS containing 0.75% bovine serum albumin (PBSA). BHK-21 cells were used to titrate infectious virus in blood and tissue samples by a standard plaque assay as described previously [11].

### 2.2. Mice

Immunocompetent Balb/c, C57BL/6, A2G and WT129 mice were obtained from Harlan (Bicester, UK). Mice with targeted genetic deletions were obtained from either Harlan (UK) or Jackson Laboratories (Bar Harbor, ME, USA): MHC class II^-/-^ mice with a mutation in the Aβ complex of the MHC-II gene resulting in near complete elimination of CD4^+^ T-cells [23]; CD8 knockout (CD8α) mice have disruption in a CD8 encoding gene, *lyt-2*, and as a result do not have CD8^+^ T-cells [24]; SCID mice [25] were on a CB-17 (Balb/c) background. All mice were infected at 4–6 weeks of age. Mice were sampled at different times post-infection (p.i.), as indicated in the results section. All breeding and animal experiments were approved by The University of Edinburgh Ethical Review Committee and carried out under the authority of UK Home Office Project License PPL 60/411, granted 25 May 2010. All animals were kept in HEPA filtered, individually ventilated cages, with environmental enrichment, 12 h light dark cycle and food and water *ad libitum*.

### 2.3. Production of Hyperimmune (HI) Serum 

Forty Balb/c mice, age 4–6 weeks, were inoculated (day 0) with 5 × 10^3^ pfu of SFV A7(74) and boosted with repeat inoculations at 14 and 21 days. As a specificity control, 40 Balb/c mice were inoculated with 5 × 10^3^ pfu of the BeAn strain of Theiler’s murine encephalomyelitis virus (TMEV) and boosted with repeat inoculations at 14 and 21 days. At day 28 mice were euthanised and sera were collected from both groups, pooled and characterized by ELISA. Heparinised blood was separated by centrifugation, plasma pooled and stored at –20 °C. Microtitre plates (4 HBX, Thermo Electron Corp., Waltham, MA, USA) were coated overnight at 4 °C with band purified SFV A7(74) diluted in NaHCO_3_ pH 9.6 buffer at 1:800 dilution. All samples were assayed in triplicate. Plates were washed 3 times with PBS and 0.05% Tween 20 (PBST-Sigma, St. Louis, MO, USA) and blocked with neat CAS block (Sigma) for 10 min at room temperature. Plates were washed, diluted serum samples were added along with relevant controls: no serum; anti-TMEV serum and immune serum. The microtitre plate with sera was incubated at room temperature for 90 min and then washed as before. Horseradish peroxidase conjugated secondary antibody was added at a 1:500 dilution and incubated for 2 h at room temperature followed by a wash. Colour development was determined by adding 100 μL of tetramethyl benzidine (Sigma) substrate for 15 min, development was stopped with 0.5 M hydrochloric acid and plates were read at 450 nm on a microplate reader (Model MRX, Dynex Technologies, Chantilly, VA, USA). Serum was also prepared from Balb/c mice given a single inoculation of SFV and sampled at d7 pi (SFV d7 serum). For serum isotyping, plates were coated with virus and blocked with neat CAS block. Bound test antibodies were detected with secondary antibodies to individual immunoglobulin isotypes (incubation 30 min) followed by detection as described above.

Adoptive transfer of antibody. SCID mice, age 4–6 week, were inoculated i.p. with 5 × 10^3^ pfu SFV A7(74). At d4 p.i., groups of SCID mice received a passive transfer of either, 0.1 mL of HI SFV serum, 0.1 mL of HI TMEV serum or PBS. Passive transfers were repeated every 3 days until d19 p.i. At post-infection days 21, 42 and 63 mice were perfused and sampled.

### 2.4. RNA Extraction

Half brain specimens were submerged in RNA stabilization reagent, RNA*later* (Sigma). RNA was extracted using the RNeasy Lipid kit (Qiagen, Manchester, UK) according to manufacturer’s instructions including the optional on-column DNase step. RNA was stored at −80 °C. RNA quantity was determined by spectrophotometry (NanoDrop, Thermo Fisher Scientific, Waltham, MA, USA) and analysed for quality on the Agilent RNA 6000 Nano Assay.

### 2.5. Quantitative RT-PCR

cDNA was produced from total RNA using Superscript II RNase H Reverse Transcriptase and amplified with primers targeting a 173 bp fragment of the E1 structural gene of SFV. The sequence of the primers was: 5′-CGC ATC ACC TTC TTT TGTG-3′ for the forward primer and 5′-CCA GAC CAC CCG AGA TTT T-3′ for the reverse primer. Real-time PCR was performed using FastStart DNA Master SYBR Green I kit (Roche, Basel, Switzerland). An initial denaturation step at 95 °C for 10 min was followed by 40 cycles of amplification. Each cycle comprised denaturation at 94 °C for 10 s, annealing at 62 °C for 5 sec and extension at 72 °C for 10 sec. The Tm of the 173 bp product was approximately 88.5 °C. For the quantitative PCR standards, an in vitro transcript from the pGEM1-SFV cDNA plasmid containing the structural genes of SFV was transcribed using a Promega RiboMax kit (Southampton, UK). Serial dilutions of the plasmid were made to produce a standard curve. 

### 2.6. Cyclophosphamide Treatment

Four to 6 week old mice were infected i.p. with a single dose of 5 × 10^3^ pfu SFV A7(74) and allowed to recover. Forty or 80 weeks after infection, mice were given a single dose of 200 mg/kg cyclophosphamide or PBS. At d4, d7 and d14 post-immunosuppression, brains were harvested and virus infectivity titers determined.

## 3. Results

### 3.1. Antibodies Are Sufficient to Reduce Infectious Virus in The Brain to Undetectable Levels but Cannot Eliminate All Potentially Infectious Virus Material

It is well established for both SFV and Sindbis virus (SINV) that antibodies are required to reduce levels of infectious virus in the brain to undetectable during the acute phase of infection; in the absence of antibodies, infectious virus can persist in the brain for long periods [20,21,26]. However, in both SFV and SINV infections, even in immunocompetent mice, virus RNA persists in the brain for weeks or months after the acute infection and long after infectious virus is detectable [15,18]. To determine whether infectious virus in the brain would again be detectable if antibodies were removed weeks after infectious virus titers had been reduced to undetectable, we studied passive antibody transfer in SFV infected SCID mice.

**Figure 1:**
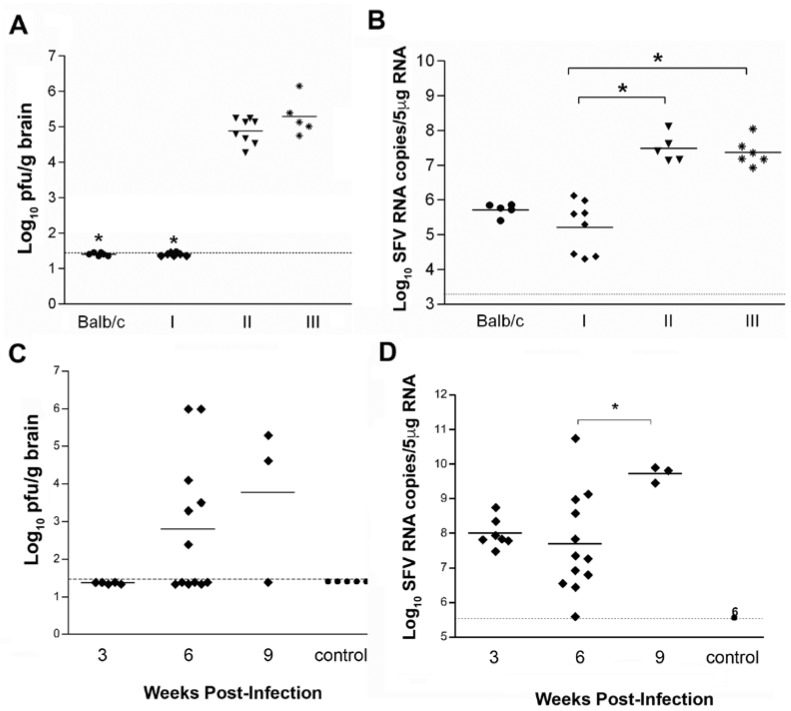
Antibodies cannot eliminate the virus from the brain in the absence of T cell immunity. (**A**) Infectious virus titres at 3 weeks post-infection in SFV infected BALB/c mouse brain; I–SFV infected SCID mice that received HI SFV serum (♦), *n* = 8; II–SFV infected SCID mice that received TMEV d28 serum (▼), *n* = 8 and III–SFV infected SCID mice that received PBS (✹), *n* = 6. * indicates significant difference (*p* < 0.01) compared to control groups (II and III). (**B**) SFV RNA copies measured by q-PCR in brain tissue at 3 weeks post-infection in SFV infected BALB/c ‘control’ mice (●), I–SFV infected SCID mice that received HI serum (♦), II–SFV infected SCID mice that received Theiler’s virus serum (▲) and III–SFV infected SCID mice that received PBS (▼). Each point represents the mean of two replicates. (**C**) Long-term virus infectivity titres in the brains of SFV infected SCID that received HI serum (♦) or control mice (●). (**D**) SFV RNA copies in the brains of SFV infected SCID mice that received HI serum (♦) or control mice (●), as measured by q-PCR. * indicates *p* < 0.05. Each point represents the mean of two replicates. Titres were measured by plaque assay in BHK-21 cells. Data were analyzed by Mann-Whitney test; dashed line indicates the limit of detection for the assay.

We first determined whether antibodies were sufficient to eliminate infectious virus if transferred to SCID mice and whether virus RNA would persist. Groups (*n* = 8) of SCID (Balb/c background) and Balb/c mice were infected i.p. with 5 × 10^3^ pfu SFV A7(74). Starting at day (d) 4 p.i., mice received a regular series of i.p. inoculations of hyperimmune (HI) serum raised against SFV A7(74), HI serum raised against Theiler’s murine encephalomyelitis virus (TMEV) or, as a second control PBS. Day 4 p.i. was chosen as the starting point for passive transfer of antisera since high titres of SFV are reproducibly detectable in the brains of SCID mice, and antibodies are first detectable in the serum of immunocompetent mice, at day 4 or 5 [20,21]. To maintain high antibody levels for weeks, mice received multiple doses of serum. To check the efficacy of the passive antibody transfers, levels of anti-SFV antibodies in the serum were determined by ELISA at d21 p.i. SCID mice that received anti-SFV HI serum had comparable titres of anti-SFV antibodies to immunocompetent Balb/c mice, whereas SCID mice that received either PBS or anti-TMEV HI serum had no detectable SFV specific antibodies. Titres of infectious virus in the brain were determined by plaque assay on d21. SCID mice that received either PBS or control anti-TMEV HI serum had high viral titres ranging from log_10_ 4.1 to 6.2 pfu/g (Figure 1A). In contrast, no virus could be detected at d21 in the brains of SCID mice (8/8) that received anti-SFV HI serum (Figure 1A). Infectious virus was also below the limit of detection in all (8/8) immunocompetent Balb/c mice. Viral RNA was isolated from the brain and levels were determined by q-PCR. Virus RNA was detectable in all groups of mice (Figure 1B). SCID mice that received anti-SFV HI serum had a significantly lower level of SFV RNA than SCID mice that received control HI serum (*p* = 0.002) or PBS (*p* = 0.004). There was no significant difference in virus RNA load between anti-SFV HI serum treated SCID and control Balb/c mice (*p* > 0.5). These data indicate that in the absence of T cell immunity, antibodies are sufficient to clear infectious virus from the brain and reduce levels of viral RNA to those observed in immunocompetent mice.

To determine whether this persisting virus RNA can again give rise to infectious virus if antibodies are removed, virus infectivity levels were determined after withdrawal of antibody passive transfers. Anti-SFV HI serum was transferred to SFV-infected SCID mice, as described above, and virus titres were determined in the brain 2 days, and 3 and 6 weeks after the last serum transfer (d21, d42 and d63 pi, respectively). At d21 pi (d2 post-serum transfer), no infectious virus could be detected in the brains of infected SCID mice (0/6) (Figure 1C). At d42 pi (3 weeks post-serum transfer) 50% (6/12) SCID mice had detectable infectious virus in the brain with virus titres ranging from log_10_ 2.2 to 6.1 pfu/g (Figure 1C). At 63 days pi (6 weeks post-serum transfer), 66% (2/3) SCID mice had detectable infectious virus in the brain. As expected, mock-infected SCID mice had no detectable virus (Figure 1C). In parallel, viral RNA levels were determined in the brain. Virus RNA remained detectable in the brains of all SFV-infected, antibody-treated SCID mice (Figure 1D). There was no significant difference in viral RNA levels at d21 and d42 post-infection, however, at d63 there were significantly higher virus RNA levels (*p* = 0.036). No RNA was detected in mock-infected SCID mice. Collectively, these results demonstrate that, in the absence of T cell immunity, antibodies alone can reduce virus infectivity levels to below the limit of detection of the infectivity assay but, as demonstrated by the continued presence of virus RNA, antibodies are not sufficient to eliminate all virus material and this virus material can reinitiate infection when antibody levels decline.

### 3.2. MHC Class II Knockout Mice Are Unable to Clear Infectious Virus from The Brain

**Figure 2:**
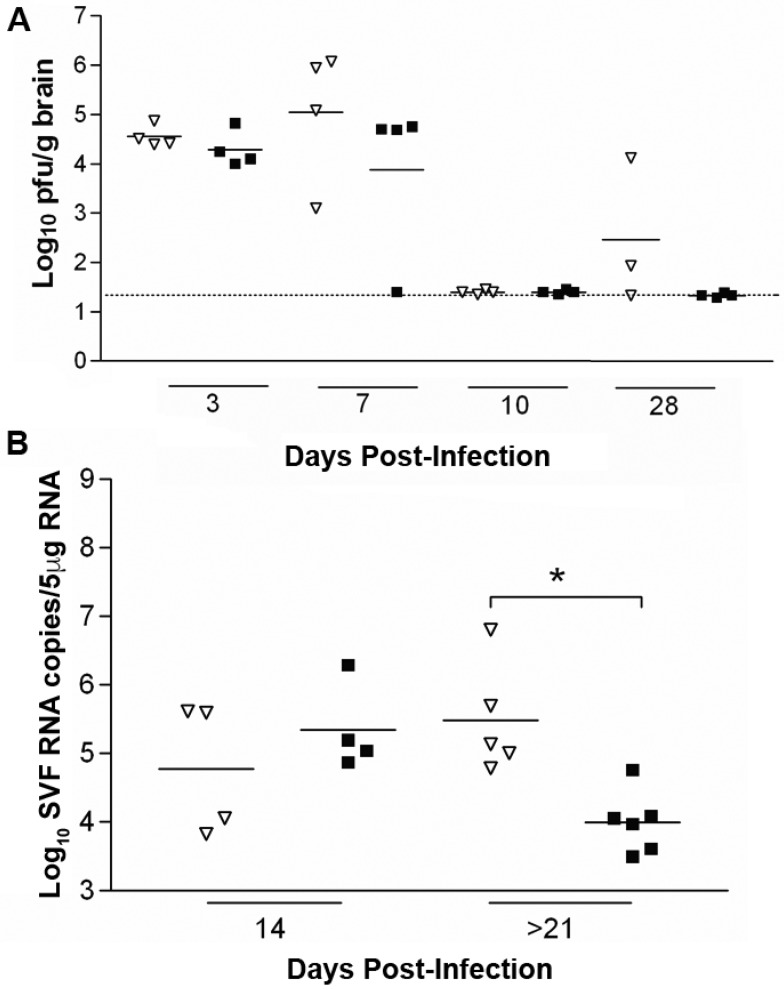
CD4^+^ T cells are required to control SFV persistence. (**A**) Infectious virus titres in the brains of SFV infected MHCII-/- mice (∇) and C57Bl/6 mice (■) at different time points post-infection. (**B**) SFV RNA copies in the brains of MHC-II^-/-^ mice (∇) and C57BL/6 mice (■), as measured by q-PCR. Dashed line indicates the limit of detection, * indicates *p* < 0.05. Data were analysed by Mann-Whitney test. <21 days post-infection is combined data from d21 and d28.

To investigate the contributions of components of cellular immunity in viral elimination from the brain, we infected mice with a disruption in the MHC class II Aβ (*H2-Ab1* gene) (MHC-II^-/-^), which do not express MHC class II and have no CD4^+^ T cells. Groups (*n* = 20) of C57BL/6 MHC-II^-/-^ and WT C57BL/6 mice were infected with 5 × 10^3^ pfu SFV A7(74) and sampled between d3 and d28 pi. MHC-II^-/-^ mice showed increased susceptibility to SFV infection with 30% mortality prior to sampling. Viral infectivity titres in the brain were examined on d3, d7, d10 and d28 pi. Virus was detectable in all (4/4) MHC-II^-/-^ mice on each of d3 and d7, but in none of the mice (0/4) on d10 (Figure 2A). Similarly, virus was detectable in all (4/4) control C57BL/6 mice on d3 and most (3/4) mice on d7, but none (0/4) of the mice on d10. MHC-II^-/-^ mice showed elevated, but not significantly different levels of infectious virus compared to control mice at d3 pi (log_10_ 4.5 and 4.3 pfu/g, respectively) and d7 (log_10_ 5 and 3.5 pfu/g, respectively). At d28 pi, infectious virus was again detectable in (2/3) MHC-II^-/-^ mice while no infectious virus was detectable in control WT mice (Figure 2A).

Levels of SFV RNA in the brain were also determined. At d10 pi, viral RNA levels were similar (log_10_ 4.8 and 5.3 copies of SFV RNA/5μg of total RNA, respectively) (Figure 2B). At d28, virus RNA levels were significantly higher in MHC-II^-/-^ mice (*p* = 0.004) compared to WT mice. Sera from both MHC-II^-/-^ and WT mice were isotyped on d10 pi. As expected, the lack of CD4^+^ T cells in MHC-II^-/-^ mice affected isotype switching and very low levels of SFV-specific antibodies, IgM and IgG2b only, were detected in these mice compared to WT mice or anti-SFV HI serum.

These data indicate that CD4^+^ T cells are required, directly or indirectly (probably for antibody isotype switching), to maintain levels of infectious brain virus below the limit of detection. This finding reinforces that from the passive antibody transfers to SCID mice; virus persist in the brain in a form that, upon decline of antibody levels, can again give rise to infectious virus. It also extends this by showing that CD8^+^ T-cells are insufficient to prevent the re-emergence of infectious virus if antibody levels decline.

### 3.3. Lack of CD8^+^ T Cells Leads to Delayed Viral RNA Clearance in The Infected Brain

**Figure 3:**
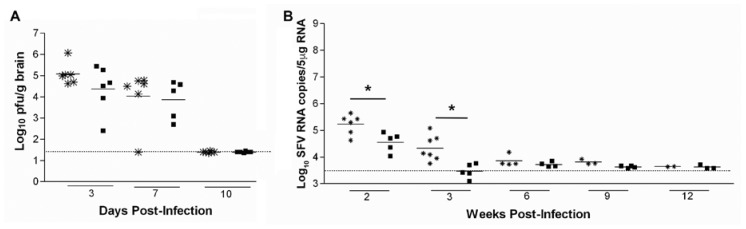
CD8^+^ T cells contribute to clearance of viral RNA in the brain. (**A**) Infectious virus titres in the brains of SFV infected CD8α mice (✹) and C57BL/6 mice (■), as measured by plaque assay on BHK-21 cells. No infectious virus was detected in 5/5 CD8α or 5/5 C57BL/6 mice at each of days 14 and 21 pi. (**B**) Long term clearance of SFV RNA in the brains of SFV infected CD8α mice (✹) and C57BL/6 mice (■), as measured by q-PCR. * indicates *p* < 0.05, data were analysed by Mann-Whitney test, dashed line indicates the limit of detection.

To investigate events in the absence of CD8^+^ T cells, levels of infectious virus and virus RNA were measured in the brains of infected CD8α mice that lack CD8^+^ lymphocytes. CD8α and C57BL/6 mice (*n* = 30) were infected with 5 × 10^3^ pfu SFV A7(74). Virus titres were determined in the brain during the first 3 weeks of infection (d3, d7, d10, d14 and d21) and levels of viral RNA were determined up to 12 weeks pi. No significant difference was observed in the rate of infectious virus clearance in the brain between CD8α and WT (C57Bl/6) mice (Figure 3A). However, the levels of viral RNA in CD8α mice were significantly higher at 2 and 3 weeks pi (*p* = 0.0303 and *p* = 0.0025, respectively), compared to WT mice (Figure 3B). At 6, 9 and 12 weeks pi, all mice in both groups had low, but persistent levels of virus RNA in the brain (Figure 3B). CD8α mice had a slightly higher, but not significantly different mean virus RNA load at each of these time points compared to WT mice. These data indicate that that CD8^+^ T cells are not required to clear infectious virus from the brain, but that they contribute to reducing the levels of virus RNA.

### 3.4. In Immunocompetent Mice, Infectious Virus Can Also Be Recovered Many Months after Infection

**Figure 4:**
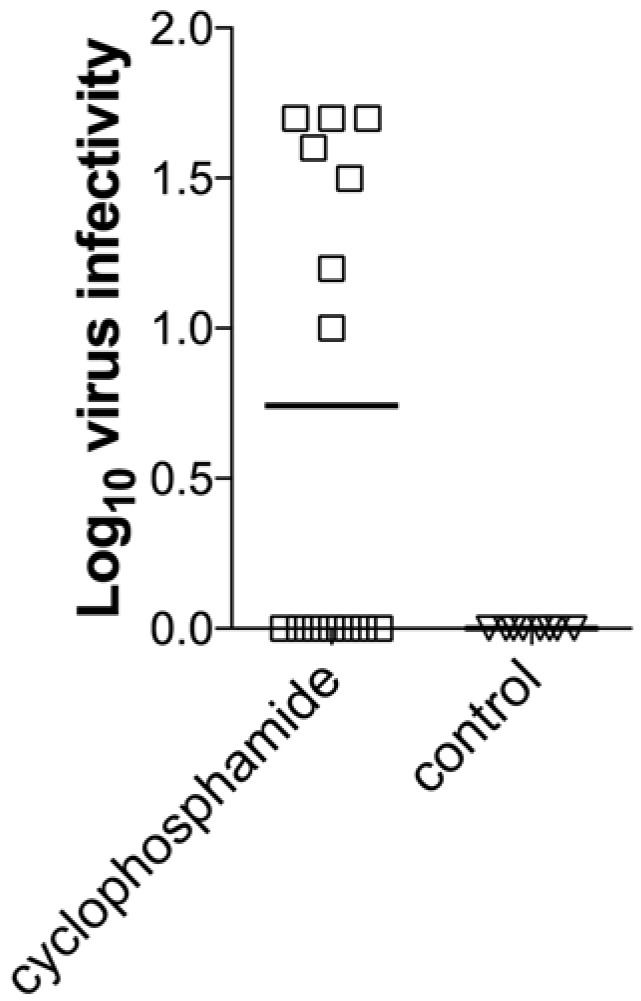
Treatment of mice with cyclophosphamide results in recrudescence of SFV. Infectivity assay on brains from SFV-infected and asymptomatic mice treated with cyclophosphamide (200 mg/kg single dose i.p.) or PBS 40-80 weeks post-infection. Combined data are plotted for immunosuppressed group (*n* = 14) with virus detected on d4 (3 mice), d7 (3 mice) and d14 (1 mouse). No virus was detectable in the control mice (*n* = 8).

To determine whether infectious virus could be recovered from immunocompetent mice, weeks or months after infection, immunocompetent mice were infected with SFV A7(74) and then given a single high dose (200 mg/kg) of the immunosuppressive drug cyclophosphamide or, as a control, PBS, 40 or 80 weeks after infection. At d4, d7 and d14 post-immunosuppression, brain virus infectivity titers were determined. 50% (7/14) of immunosuppressed mice had detectable infectious virus, whereas none (0/8) of the controls had detectable virus (Figure 4). This ability to recover infectious virus up to 18 months after initial infection in immunocompetent mice, indicates that this virus can persist in the mouse brain essentially for life (the life span of a laboratory mouse is approximately 2 years).

## 4. Discussion

Infections with RNA viruses, other than retroviruses, most commonly cause a quickly resolving acute disease with clearance of infectious virus within a couple of weeks. However, RNA viruses can persist for long periods of time and this is more likely to occur in sites with specialised immune responses such as the gonads, the eye, the joints or the brain. Experimentally, many RNA viruses can persist in the mouse brain, either in immunocompetent mice, as is the case with lymphocytic choriomeningitis virus [27], or in immunocompromised mice, as is the case with SFV A7(74) in SCID or athymic *nu/nu* mice [20,21]. Human CNS diseases such as multiple sclerosis, post-polio syndrome and subacute sclerosing panencephalitis have been linked to persistent RNA virus infections [28,29,30].

In the highly specialised environment of the CNS, where many cells are not renewable and must survive a life-time, cell death as a response to infection is limited [8,31,32]. In the CNS, the induction of, and actions of, cytotoxic T cells, antibodies, complement and inflammation are all highly regulated [26]. The longevity of neurons, their resistance to induction of cell death and the strong control of CNS immune responses provide an environment in which non-destructive RNA viruses can persist.

In the case of immunocompetent mice infected with SFV A7(74), we have previously shown that infectivity in the brain is detectable for only 7 to 10 days p.i. but that virus RNA is detectable for at least 8 weeks [11,15]; we have confirmed this here. Presence of alphavirus RNA in the brain of immunocompetent mice months after infection has also been reported for the M9 strain of SFV and the AR339 strain of SINV [18,33]. We have also previously shown that in the brains of athymic (*nu/nu*), SCID or B-cell deficient μMT mice, infectious SFV A7(74) virus is detectable for months and that virus persists in neurons and oligodendrocytes without causing any apparent damage to these cells [11,15,20,21].

Single passive transfers of immune serum or monoclonal antibodies into immunocompromised animals have previously been shown to rapidly clear, or significantly reduce, alphavirus burden [20,21,22,34,35,36]. In the case of SINV, clearance of infectious virus from the brains of SCID mice by a single dose of hyperimmune serum was followed weeks later by the re-emergence of infectious virus in the brain [18]. This study did not address whether sustained high levels of anti-viral antibodies would eliminate infectious virus. In the present study, we used multiple dose passive transfer of high titre neutralizing anti-SFV HI serum over a two-week period. This reduced infectious virus to levels below the limit of detection and reduced levels of virus RNA to the low levels observed in immunocompetent controls. However, 3 to 6 weeks after the serum was withdrawn, infectious virus was again detectable in the brain, indicating that even sustained high levels of neutralizing antibodies are not sufficient to eliminate from the brain virus material capable of forming infectious virus.

Mice lacking CD4^+^ T cells initially clear infectious SFV at the same rate as WT controls, this corroborates a study on the early stage of infection in SINV infected CD4 knockout mice [37]. Here we extend these observations by demonstrating that in CD4 null mice, brain virus RNA levels remain at a significantly higher level than in WT mice, and that at 4 weeks post-infection infectious virus can again be detected. This shows that CD4^+^ T cells play a role in maintaining undetectable levels of infectious virus. It is most likely that this represents CD4^+^ T-cell help for antibody isotype switching in B-cells. In the absence of this help, early levels of IgM and IgG2b decline, other antibodies isotypes are not generated and infectious virus re-emerges. However, CD4^+^ cells may also contribute to the control of virus through other mechanisms. B-cell deficient (μMT) mice infected with the related alphavirus Venezuelan equine encephalitis virus develop a persistent brain infection. Depletion of CD4^+^ T cells increased viral brain titers and disease severity indicating that non-antibody mediated mechanisms of CD4^+^ T-cell mediated infectious virus control are also functioning [38]. Studies with SINV show that CD4^+^ T-cell production of IFN-γ plays a role in maintaining brain infectious virus levels undetectable [39,40].

Our data from MHC-II^-/-^ mice could indicate that CD8^+^ T cells are insufficient to control infectious SFV in the brain, at least in the absence of CD4^+^ T cell help. Therefore, we investigated infection in CD8α knockout mice to determine whether these cells played any role in virus clearance or persistence. CD8α knockout mice cleared infectious virus at the same rate as WT mice, however the rate of virus RNA clearance was significantly slower. These data are consistent with results for SINV infection [41]. The initial difference in virus RNA clearance did not persist at later time points (weeks 6, 9 and 12). After resolution of the acute infection, CD8α knockout mice had no detectable infectious virus showing that these cells are not required to maintain levels of virus infectivity undetectable. A more prominent protective role for CD8^+^ T cells is apparent with other neurotropic RNA viruses [42,43,44,45]. In case of alphaviruses, it appears that these cells facilitate reduction of the virus RNA load, but their function or lack thereof, does not significantly affect clearance of infectious virus from the brain. However, in the case of SFV A7(74), CD8^+^ T destroy virus infected oligodendrocytes leading to lesions of immune-mediated demyelination [14].

Previous attempts to recover SINV from the brain following treatment with immunosuppressive agents were unsuccessful [18]. However, cyclophosphamide treatment of mice infected with the neurotropic flaviviruses Japanese encephalitis virus [46] and West Nile virus [47] did result in the renewed detection of infectious virus; though in these studies immunosuppression was carried out early after infection. Severe infections including apparent RNA virus reactivation have been reported in immunocompromised patients [48], and there is a recent report of a fatal case of Eastern equine encephalitis in a patient undergoing immunosuppressive therapy [49]. Our results show for the first time that many months after initial infection, infectious virus can be isolated from the brains of immunocompetent mice following immunosuppression.

In immunocompetent mice, both SFV and SINV induce florid CNS, predominantly mononuclear cell, inflammatory responses. These subside with clearance of infectious virus but low levels of inflammatory cells, predominantly B220^+^ B-cells, and relatively high levels of anti-viral intrathecal antibodies are maintained at a steady level for months [16,17,19]. This maintenance of the anti-viral response in the absence of detectable infectious virus has long been enigmatic. However, it is completely consistent with our demonstration, in both SCID and immunocompetent mice, that persistent brain virus RNA can again give rise to infectious virus if antibody levels decline. The nature of the virus material that persists in the brain is not clear. The virus RNA that is detectable by PCR could represent a mixture of RNA species, including degraded non-functional RNA. However, for infectious virus to be regenerated it is likely that full-length genomic RNA is present. This could be in the form of virions, either within cells or outside them, intracellular virus replication complexes or aggregates of virus RNA.

Many CNS cells, particularly neurons are long-lived and in the absence of immune responses SFV A7(74) can persist in these cells for many months without causing any apparent damage [8,10,21]. This has also been established for other RNA viruses [27]. In the case of SFV A7(74), virus replication is restricted in mature neurons, few virions are produced, the virus replication complexes characteristic of other cells and immature neurons are not formed and virus RNA accumulates in large aggregates [11]. In long-lived neurons, which do not undergo apoptosis upon infection, these aggregates of virus RNA could be stable. They could also give rise to low levels of virions. Other cell types could also be producing infectious virus. This persistent infection continues to stimulate brain immune responses, resulting in the continual low level pleocytosis and antibody production. This could result from intracellular virus RNA activating cellular defences, virus protein production within or on the surface of cells or the production of low levels of virions within, or released out of, infected cells. Whatever the mechanism by which the immune response is perpetuated, antibodies within the brain could function to prevent virion production [22] or to neutralise aggregates of virus material or formed virions. While antibody neutralisation of infectious material may be continuous in the tissue there is also the possibility that infectivity is undetectable as a result of neutralisation at the time of tissue homogenisation prior to assay. Whatever the nature of the infectious material and the mechanism by which infectivity is undetectable for long periods, it is now clear that SFV can persist essentially for life in the mouse brain in a form that can again give rise to infectious virus if antibody levels decline.
